# Supplementary material for: A Gene from Ganoderma lucidum with Similarity to nmrA of Filamentous Ascomycetes Contributes to Regulating AreA
Source: J Fungi (Basel). 2023 Apr 26;9(5):516. doi: 10.3390/jof9050516 (PMC10219401; doi:10.3390/jof9050516)
Supplement: Supplementary file 1 [file jof-09-00516-s001.zip › jof-2339441-supplementary.pdf]

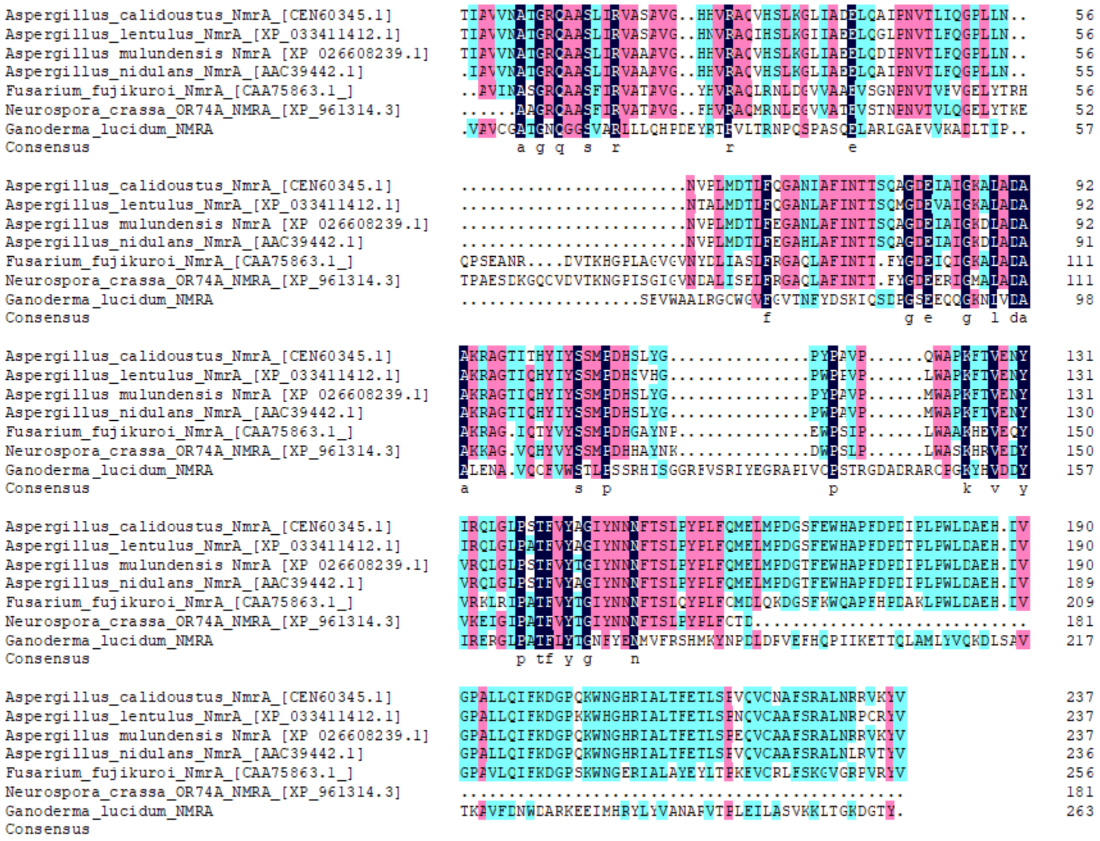

Figure S1. Homologous comparison of the conserved domain of NmrA in filamentous ascomycetes and *G. lucidum*. These filamentous ascomycetes include *A. calidoustus* [CEN60345.1], *A. lentulus* [XP_033411412.1], *A. mulundensis* [XP_026608239.1], *A. nidulans* [AAC39442.1], *F. fujikuroi* [CAA75863.1], *N. crassa* OR74A [XP_961314.3].

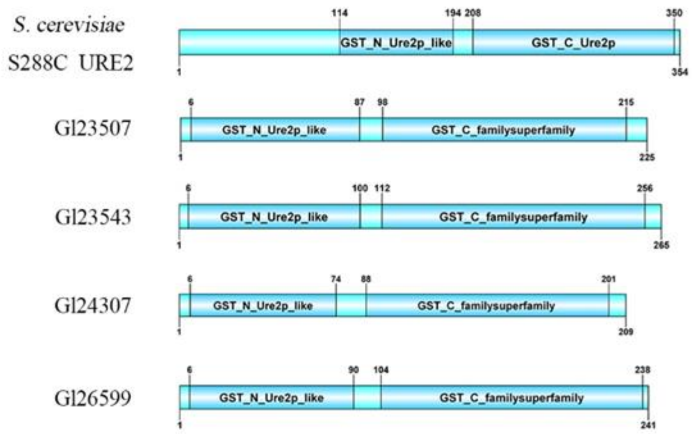

Figure S2. Analysis of conserved domains of the Ure2 in *S. cerevisiae* and four suspected homologous Ure2 in *G. lucidum*.

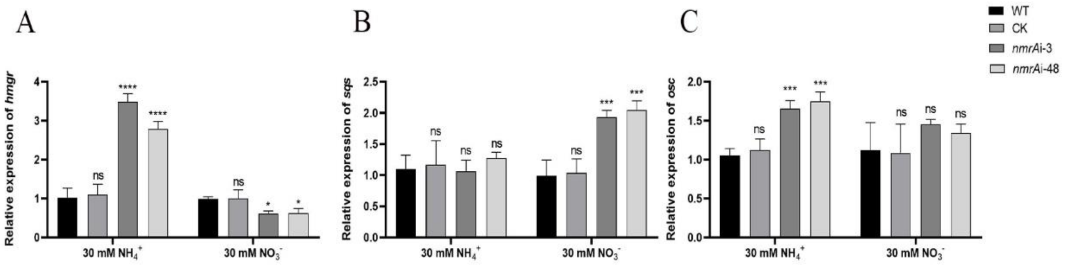

Figure S3. Expression levels of GA synthesis genes (*hmgr*, *sqs* and *osc*) in the WT, CK, and *nmr* silenced strains under the ammonium and nitrate conditions. All data are presented as means ± SDs (n = 3). Statistical significance is represented by asterisks (one way ANOVA, * $p < 0.05$, ** $p < 0.01$, *** $p < 0.001$, **** $p < 0.0001$, ns: no significance).
